# Monitoring NAD(H) and NADP(H) dynamics during organismal development with genetically encoded fluorescent biosensors

**DOI:** 10.1186/s13619-021-00105-4

**Published:** 2022-02-01

**Authors:** Ting Li, Yejun Zou, Shuning Liu, Yi Yang, Zhuo Zhang, Yuzheng Zhao

**Affiliations:** 1 Optogenetics & Synthetic Biology Interdisciplinary Research Center, State Key Laboratory of Bioreactor Engineering, Shanghai Frontiers Science Center of Optogenetic Techniques for Cell Metabolism, East China University of Science and Technology, 130 Mei Long Road, Shanghai, 200237 China; 2 Shanghai Key Laboratory of New Drug Design, School of Pharmacy, East China University of Science and Technology, 130 Mei Long Road, Shanghai, 200237 China; 3 Research Unit of New Techniques for Live-cell Metabolic Imaging, Chinese Academy of Medical Sciences, Beijing, China

**Keywords:** Cell metabolism, NAD(H) and NADP(H), Genetically encoded fluorescent sensors, Real-time monitoring, Organismal development

## Abstract

Cell metabolism plays vital roles in organismal development, but it has been much less studied than transcriptional and epigenetic control of developmental programs. The difficulty might be largely attributed to the lack of in situ metabolite assays. Genetically encoded fluorescent sensors are powerful tools for noninvasive metabolic monitoring in living cells and in vivo by highly spatiotemporal visualization. Among all living organisms, the NAD(H) and NADP(H) pools are essential for maintaining redox homeostasis and for modulating cellular metabolism. Here, we introduce NAD(H) and NADP(H) biosensors, present example assays in developing organisms, and describe promising prospects for how sensors contribute to developmental biology research.

## Background

Environmental nutrients and cellular metabolism are crucial for organismal development (Aplin et al. 2020). During embryonic development, cellular metabolism is dynamically programmed; for example, pyruvate is the main nutrient supporting development from the zygote to morula stage, and glucose plays a central role in nutrition during the ensuing morula-to-blastocyst transition (Brown and Whittingham 1991). The nutritional functions of pyruvate and glucose are not interchangeable, although pyruvate is just the glycolytic product of glucose, thereby highlighting the highly programmable requirements of nutrients and metabolism for development. The importance of developmental metabolism is clearly evidenced by broad developmental defect spectra in babies with inborn error of metabolism (Kruszka and Regier 2019).

**Fig. 1:**
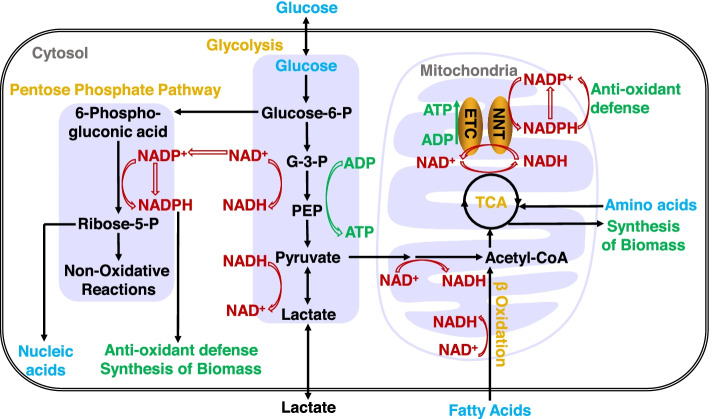
NAD(H) and NADP(H) play a vital role in cellular metabolism. NAD(P)(H) are key coenzymes for cellular metabolism. Cytosolic NADH is produced during glycolysis, which is either consumed during conversion of pyruvate into lactate or transported into mitochondria by reducing equivalent shuttle pathways. Mitochondrial NADH is generated by oxidation of pyruvate and fatty acids and in the tricarboxylic acid cycle (TCA), and it is oxidized back into NAD^+^ by the electron transfer chain (ETC). Glycolysis and mitochondrial respiration supply energy production. NADPH is mainly produced by the pentose phosphate pathway and other metabolic enzymes whose isoforms exist in both the cytosol and mitochondria, and it is consumed for the synthesis of biomass and antioxidant defense responses. The NAD(H) and NADP(H) pools connect through NAD^+^ kinase (NADK) and nicotinamide nucleotide transhydrogenase (NNT). Abbreviations: glucose-6-P, glucose-6-phosphate; G-3-P, glyceraldehyde-3-phosphate; PEP, phosphoenolpyruvate; ribose-5-P, ribose 5-phosphate

The human metabolome consists of more than 4100 metabolites(Brunk et al. 2018). Among them, core coenzymes I and II, i.e., nicotinamide adenine dinucleotides (NAD^+^ and NADH) and their phosphorylated forms (NADP^+^ and NADPH), are involved in a large number of metabolic reactions and thus have a systematic impact on the entire metabolic network (Fig. 1). In eukaryotic cells, there are two main pyridine dinucleotide pools: cytosol and mitochondria. The cytosolic free NAD^+^ concentration is approximately 50-110 μM and is usually 60-1000-fold higher than NADH(Cambronne et al. 2016; Sallin et al. 2018). Mitochondrial free NAD^+^ is approximately 230 μM, and the ratio of NADH/NAD^+^ reaches approximately 0.1 to 1(Cambronne et al. 2016; Sallin et al. 2018). The free NADPH concentration is approximately 3 μM in the cytosol and 37 μM in mitochondria, and NADPH is dominant over NADP^+^, with an NADPH/NADP^+^ ratio ranging from 15 to 333(Hedeskov et al. 1987; Tao et al. 2017; Veech et al. 1969). The cytosolic and mitochondrial NAD(H) and NADP(H) pools are relatively independent of each other. However, NADH can be transported between these compartments via the malate-aspartate or glycerol phosphate shuttle, and the mitochondrial NAD^+^ transporter SLC25A51 has recently been identified(Kory et al. 2020; Luongo et al. 2020).

NAD(H) plays a fundamental role in redox homeostasis and energy metabolism. Since NAD(H) participates in more than one hundred redox reactions as electron carriers, the ratio of NAD^+^ to NADH is a commonly used biochemical index for cellular redox status. Cytosolic NADH is primarily generated by glycolysis, while it can be consumed either during the reduction of pyruvate to lactate or transport into mitochondria (Fig. 1). In mitochondria, NADH is generated via oxidation of pyruvate and fatty acids and the tricarboxylic acid (TCA) cycle, and it is consumed for ATP production via mitochondrial respiration. As NADH oxidized form, NAD^+^ is mainly synthesized through three pathways, including the salvage pathway from the precursor nicotinamide, nicotinamide mononucleotide, nicotinamide riboside or dihydronicotinamide riboside (Verdin 2015); the Preiss-Handler pathway from nicotinic acid (Verdin 2015); or the de novo pathway from tryptophan (Verdin 2015), aspartate (Begley et al. 2001), and chorismate (Ding et al. 2021). In addition, NAD^+^ is consumed by a few regulatory enzymes, including SIRT, PARP, ART, CD38, and SARM1(Covarrubias et al. 2021), and is also involved in the 5′ cap modification of mRNAs (Kiledjian and Eukaryotic, 2018). Therefore, NAD^+^ plays important roles in cell metabolism, cell signaling, and gene expression regulation (Chini et al. 2021).

Although structurally similar to NAD(H), NADP(H) has distinct biochemical functions. NADP(H) provides fundamental reducing power for biosynthetic reactions and antioxidant functions. Cytosolic NADPH is mainly produced via the pentose phosphate pathway in mammalian cells. In addition, a few enzymes, including isocitrate dehydrogenases, malic enzymes, and methylenetetrahydrofolate dehydrogenases, contribute to the production of NADPH in both the cytosol and mitochondria (Fan et al. 2014) (Fig. 1). NADPH maintains cellular redox homeostasis via two enzymatic antioxidant defense systems: the glutathione system (GSH/GSSG) and the thioredoxin system (Trx-SH/Trx-SS); NADPH consumption also occurs in the biosynthetic process of fatty acids, deoxyribonucleotides and some amino acids. Moreover, NADPH is used as a substrate of NADPH oxidase that produces radical oxygen species on the plasma membrane (Bedard and Krause 2007). One direct link between NAD(H) and the NADP(H) pool is phosphorylation of NAD^+^ into NADP^+^ mediated by NAD^+^ kinase in the cytosol or mitochondria (Zou et al. 2018), and the other occurs via transdehydrogenase in mitochondria, which converts NADH and NADP^+^ to NAD^+^ and NADPH under a proton gradient (Gameiro et al. 2013; Pollak et al. 2007).

Therefore, NAD(H) and NADP(H) integrate inputs from a few metabolic pathways and exert their influences on various metabolic activities. While they both globally report the redox status of cells, NAD(H) and NADP(H) reflect the integrated activities of energy and anabolic metabolism, respectively. Since these metabolic activities are closely associated with organismal development, monitoring NAD(H) and NADP(H) levels shed light on how cellular metabolism is weaved into the developmental program. To date, however, little is known about NAD(H) and NADP(H) dynamics during developmental stages. A main hurdle may be that it lacks an ideal assay for them until the development of genetically encoded fluorescent sensors, which revolutionize in situ metabolic research.

## Methods for NAD(H) and NADP(H) analysis

A number of NAD(H) and NADP(H) assays have been established. Biochemical methods, such as chromatography and mass spectrometry, are based on cellular lysis and are incompetent for living cell measurement. NADH and NADPH have autofluorescence with a 340-nm excitation maximum and a 460-nm emission maximum. Based on the optical properties, NAD(P)H autofluorescence has been studied by single-photon or multiphoton excitation to monitor metabolic states in living cells or in vivo for many years (Eto et al. 1999; Kasischke et al. 2004; Mayevsky and Rogatsky 2007). Unfortunately, NAD(P)H autofluorescence is limited by low sensitivity and cell injury caused by ultraviolet irradiation (Meleshina et al. 2017). Even worse, it is difficult to distinguish NADH and NADPH due to their similar spectra (Lowry et al. 1957). A previous study with fluorescence lifetime imaging (FLIM) reported that NADH and NADPH could be differentiated on the basis of a simple assumption that bound NADH and bound NADPH possess different fluorescence lifetimes inside the cell; however, FLIM is not technically simple for most laboratories (Blacker et al. 2014). It’s worth noting that the two-photon FLIM techniques eliminate the problem with UV irradiation and exhibit considerable penetration depth. Unlike NADH and NADPH, their oxidized counterparts NAD^+^ and NADP^+^ have no intrinsic fluorescence. In situ magnetic resonance imaging technology can dynamically detect NAD^+^ in the human brain using ^31^P spectroscopy, but it is too complicated to use for broad research (Lu et al. 2016). Two semisynthetic fluorescent probes based on fluorescence resonance energy transfer (FRET) have been developed to spatiotemporally monitor NAD^+^ and NADP(H) levels in live cells, including NAD-Snifit and NADP-Snifit, which are designed by fusing human sepiapterin reductase (SPR) with two self-labeling proteins—Halo-tag and SNAP-tag—as a FRET pair (Sallin et al. 2018). NAD-Snifit and NADP-Snifit do not self-sufficiently form intrinsic fluorophores and require exogenous dyes for labeling. Thus, the excess chemical dye has to be removed by a washing procedure, which is not only time intensive but also may render the analysis susceptible to artifacts for developmental research. Therefore, live-cell and in vivo assay methods with high spatiotemporal resolution are highly desirable for monitoring NAD(H) and NADP(H) dynamics during organismal development.

## Genetically encoded NAD(H) and NADP(H) sensors

When expressed in living cells or in vivo, fluorescent protein-based sensors can monitor the spatiotemporal dynamics of metabolites with high specificity and sensitivity and have become a revolutionary technology for metabolic research (Zhang et al. 2002). In the past two decades, over 200 genetically encoded fluorescent sensors have been developed for cellular metabolites, messengers, and conditions (De Michele et al. 2014). These biosensors generally consist of two basic components: substrate-binding proteins and one or two fluorescent proteins. Various transcription factors and regulatory proteins specifically sense intracellular biomolecules from bacteria to mammals. Substrate-sensing proteins often trigger conformational changes upon biomolecule binding, which induces fluorescence changes in fluorescent proteins. Fluorescence can be readily measured by routine instruments such as plate readers, flow cytometry or fluorescence microscopy.

To date, there are nine genetically encoded fluorescent sensors available for pyridine dinucleotides: the NAD^+^ sensors LigA-cpVenus (Cambronne et al. 2016) and FiNad (Zou et al. 2020); the NADH sensor Frex (Zhao et al. 2011); the NAD^+^/NADH ratio sensors Peredox (Hung et al. 2011), RexYFP (Bilan et al. 2014), and SoNar (Zhao et al. 2015; Zhao et al. 2016b); the NADP^+^ sensors Apollo-NADP^+^(Cameron et al. 2016) and NADPsor (Zhao et al. 2016a), and the NADPH sensor iNap (Tao et al. 2017; Zou et al. 2018) (Table 1). Fluorescent biosensors must meet several criteria for live-cell and in vivo developmental studies, including high specificity, large responsiveness, appropriate affinity, strong brightness, and ratiometric readout, which allows reliable and convenient capture of subtle changes in physiological contexts. The majority of them can hardly meet these requirements due to their poor selectivity, relatively small dynamic response (i.e., RexYFP and Apollo-NADP^+^ sensors), inappropriate affinity (i.e., Peredox and NADPsor sensors), weak fluorescence (i.e., Frex sensor), or intensiometric readout (i.e., Peredox sensor) (Table 1).

**Table 1:** Genetically encoded fluorescent sensors for NAD(H) and NADP(H)

	Species sensed	Affinity	Dynamic Changes	Sensor type	pH sensitivity	Brightness in cells	Reference
**Frex**	NADH	${\mathrm K}_{\mathrm{NADH}}:\;\sim3.7\;\mathrm{\upmu{M}}$	800%	Ratiometric	Sensitive	Weak^**f**^	(Zhao et al. 2011)
**LigA-cpVenus**	NAD^+^	${\mathrm{K}}_{\mathrm{NAD}^+}:\;\sim\:65\:\mathrm{\upmu M}$	100%	Ratiometric	Sensitive	N.D.	(Cambronne et al. 2016)
**FiNad**	NAD^+^/AXP^a^	${\mathrm{K}}_{\mathrm{NAD}^+}:\;\sim1300\;\mathrm{\upmu M}^\mathrm{b}$	700%	Ratiometric	Sensitive	N.D.	(Zou et al. 2020)
**Peredox**	NADH/NAD^+^	$\mathrm{K}_{\mathrm{NAD}^+/\mathrm{NADH}}:\;\sim330$	150%	Intensiometric	Resistant	Moderate^**f**^	(Hung et al. 2011)
**RexYFP**	NADH/NAD^+^	${\mathrm{K}}_\mathrm{NADH}:\;\sim0.18\;\mathrm{\upmu M}$	50%	Intensiometric	Sensitive	N.D.	(Bilan et al. 2014)
**SoNar**	NADH/NAD^+^	${\mathrm{K}}_{\mathrm{NAD}^+/\mathrm{NADH}}:\;\sim40$	1500%	Ratiometric	Resistant^d^	Strong^**f**^	(Zhao et al. 2015)
**Apollo-NADP**^**+**^	NADP^+^	${\mathrm{K}}_{\mathrm{NADP}^+}:\;0.1-20\;\mathrm{\upmu M}$	15-20%	Ratiometric	Resistant^e^	N.D.	(Cameron et al. 2016)
**NADPsor**	NADP^+^	${\mathrm{K}}_{\mathrm{NADP}^+}:\;\sim2000\;\mathrm{\upmu M}$	30%	Ratiometric	Resistant	N.D.	(Zhao et al. 2016a)
**iNap1-4**	NADPH	${\mathrm{K}}_\mathrm{NADPH}:\;\sim2-120\;\mathrm{\upmu M}^\mathrm{c}$	500-1000%	Ratiometric	Resistant^d^	Strong^**f**^	(Tao et al. 2017)

N.D., not determined

^a^AXP denotes ADP and ATP

^b^It is measured in the presence of 1 mM AXP

^c^NADPH sensors iNap 1–4 have different affinities with *K*_d_ values of ∼2.0 μM, ∼6.0 μM, ∼25 μM, and ∼120 μM, respectively

^d^SoNar’s and iNap’s fluorescence excited at 420 nm, dynamic range, ${\mathrm{K}}_{\mathrm{NAD}^+/\mathrm{NADH}}$ and K_NADPH_ are pH resistant

^e^Venus-tagged Apollo-NADP^+^ sensor was pH resistant in the pH range of 7.25–8, but showed a progressively decreasing dynamic range below pH 7.25

^f^The data come from side-by-side comparison studies (Tao et al. 2017; Zhao et al. 2015)

**Fig. 2:**
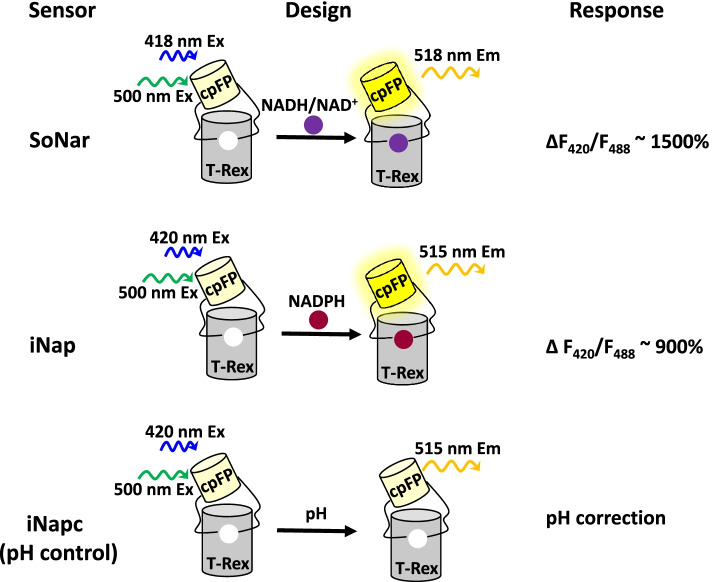
The design of NADH/NAD^+^ sensor SoNar and NADPH sensor iNap. Both SoNar and iNap are cpYFP-based single fluorescent protein sensors. The control sensor iNapc has a similar response to pH as SoNar and iNap but respond to neither NAD(H) or NADPH. For SoNar and iNap, fluorescent protein cpYFP was inserted into a monomer of NAD^+^ (NADH)-binding bacterial protein T-Rex. Binding of NADH (and NAD^+^) and NADPH induces changes in protein conformation and fluorescence of SoNar and iNap sensors, respectively

The NADH/NAD^+^ ratio sensor SoNar and the NADPH sensor iNap are superior with regard to the above criteria and are chosen to demonstrate how sensors are utilized to dynamically monitor metabolic states in living cells and live mice (Zhao et al. 2015). SoNar was designed by inserting circularly permutated yellow fluorescent protein (cpYFP) into the truncated *Thermus aquaticus* T-Rex protein (Fig. 2). The sensor has two excitation peaks, which enable an intrinsically ratiometric measurement. SoNar responds to the NAD^+^/NADH ratio but does not depend on either individual NAD^+^ or NADH concentrations alone. It exhibits a large dynamic response up to 1500% and high brightness (Zhao et al. 2015). The NADPH family sensor iNap was generated through rational design-directed protein engineering that switches SoNar’s substrate selectivity from NADH to NADPH (Tao et al. 2017), and it maintains the most superior properties of SoNar (Fig. 2). The NADPH family sensor iNap has four members with different *K*_*d*_ value, covering the most physiological concentration of NADPH in cells. The iNap1 member responds only to NADPH, with a rapid (less than 1 s) and large (900%) response. Due to their superior performances, SoNar and iNap sensors have been successfully used for metabolic studies on wound response in vivo (Tao et al. 2017), hematopoiesis (Gu et al. 2020), aging of intestinal stem cells (Morris et al. 2020), homing of leukemia-initiating cells (Chen et al. 2021; Hao et al. 2019), immortalization of neural stem cells (Bonnay et al. 2020), embryo development (Zhao et al. 2021), tumorigenesis (Ma et al. 2021), ferroptosis (Ding et al. 2020), photosynthesis and photorespiration in *Arabidopsis thaliana* (Lim et al. 2020), and so on. These studies demonstrate that SoNar and iNap sensors are not only sensitive enough to measure the physiological changes of NADH and NADPH, but also powerful for interrogating their functions. The pH fluctuation may influence the response of the two sensors, and the effect can be corrected by normalization to iNapc, which has a similar response to pH as sensors but responds to neither NAD(H) nor NADPH (Fig. 2).

## Real-time tracking of the cell cycle with SoNar and iNap1

**Fig. 3:**
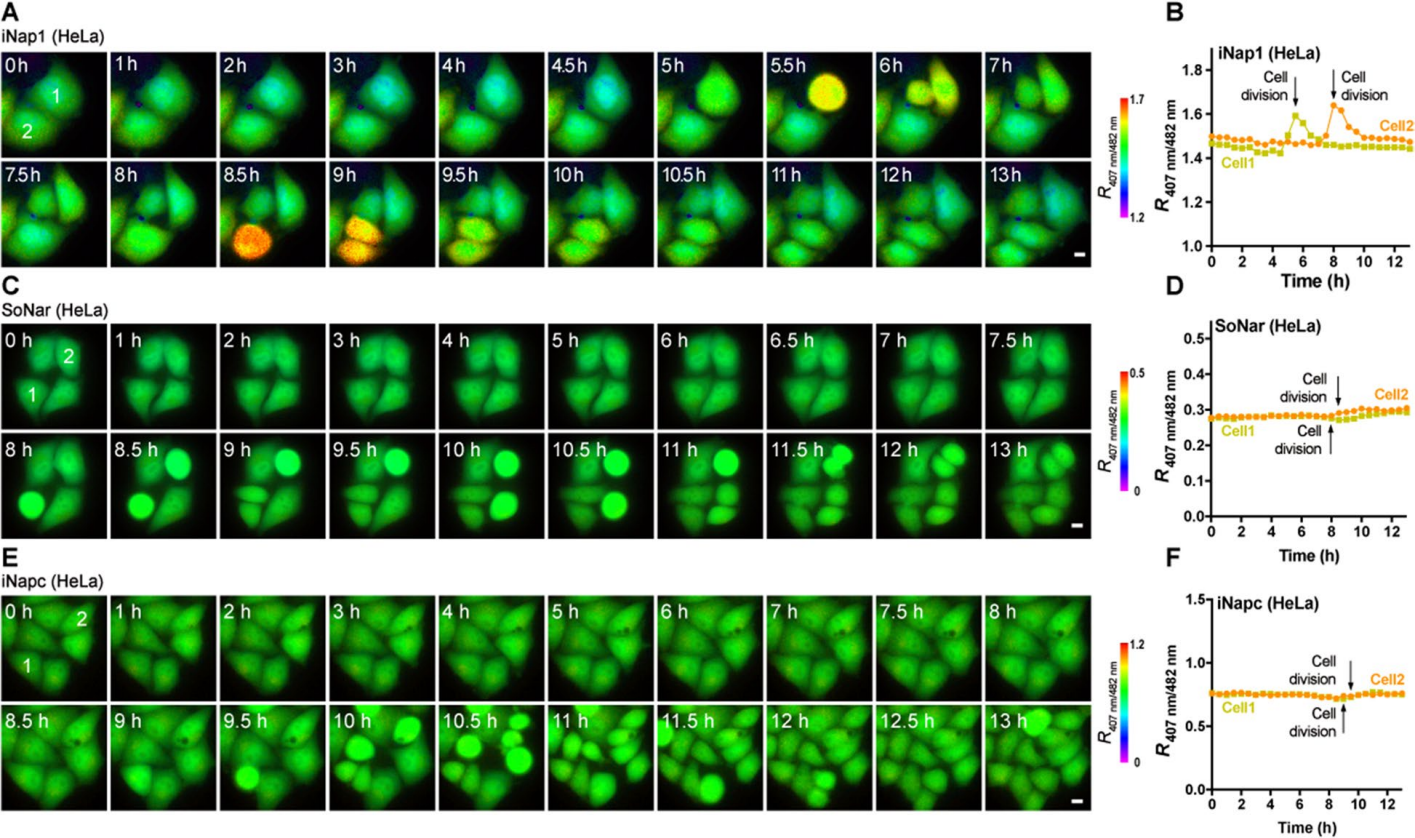
Real-time tracking NADH/NAD^+^ and NADPH dynamics during the cell cycle. Fluorescence images **A**, **C**, **E** and quantification **B**, **D**, **F** of NADPH (iNap1) **A**-**B**, NADH (SoNar) **C**-**D**, and iNapc **E**-**F** dynamics during cell division. The entire coding sequences of iNap1, SoNar, iNapc were subcloned into the vector pLVX-IRES-Puro vector for the generation of recombinant lentivirus. HeLa cells with lentiviral infection and stable expression of sensors were then seeded into glass-bottom plates. After about 24 h, the culture medium was changed into phenol-red free medium for fluorescence imaging. The fluorescence microscopy system was equipped with a Nikon Eclipse Ti-E automatic microscope, monochrome-cooled digital microscope camera (model no. DS-Qi1 Mc-U2), PFS, A Plan Apo 40 × 0.95 NA objective, a highly stable Sutter Lambda XL light source, and an on-stage CO_2_ incubator (Tokai Hit). For dual-excitation ratio imaging, 407-BP 17-nm or 482-BP 35-nm excitation filters (Semrock) and a 535-BP 40-nm emission filter altered by a Lambda 10-3 filter wheel (Sutter Instruments) were used. A perfect focus system was applied to stabilize the focal plane and high-sensitivity detectors were utilized to shorten the exposure time. The NADPH level exhibits a moderate and robust increase in the mitotic phase **A-B**, while the NADH/NAD^+^ level remains almost constant **C-D**, as well as the iNapc fluorescence **E-F**. All fluorescence images were pseudocolored by F_407_/F_482_ (R_407/482_). Scale bar, 10 μm. For **A-F**, adapted from the reference (Zou et al. 2018)

Cells copiously proliferate through the mitotic cell cycle during organismal development. Although it is clearly known that cyclin-dependent events play central roles in the control of cell cycle progression, it is less understood how cells regulate their metabolism to meet varying demands during different mitotic cell cycling phases, such as maintenance of redox homeostasis and buildup of proteins, lipids, and deoxyribonucleic acids. Here, we demonstrate how to utilize SoNar and iNap1 to visualize NADH/NAD^+^ and NADPH dynamics during the cell cycle progression of mammalian cells. To facilitate robust and long-term imaging, a coding DNA sequence for the SoNar or iNap sensor is delivered into HeLa cells via lentivirus infection, and HeLa cells stably expressing the sensor are then seeded into glass-bottom plates. Fluorescence imaging can be carried out either by a common automatic microscope equipped with an on-stage CO_2_ incubator or a high-content imaging system. A perfect focus system helps the long-term tracking of cells of interest, and optimization of imaging parameters can minimize phototoxicity, including light intensity, exposure time, gain, time interval and use of high-sensitivity detectors. The NADH/NAD^+^ level in HeLa cells did not show detectable changes during the cell cycle, while the NADPH level rose in the mitotic phase (Fig. 3A-D). The NADPH elevation is moderate and robust. Alterations in pentose phosphate pathway activities may account for the oscillating NADPH dynamics (Li et al. 2019; Ma et al. 2017). As a control, iNapc fluorescence did not significantly change during the cell cycle (Fig. 3E-F). This observation clearly exemplifies the power of sensors in capturing transient and subtle changes in metabolism, which can hardly be detected by other existing assays. During early development, eggs continuously cleave with pyruvate as the main nutrient until the morula stage; thus, it is intriguing to investigate whether NADPH fluctuates in this process and how it occurs. These sensors may be considered to solve these mysteries.

## Metabolic imaging of the embryonic development of zebrafish with SoNar and iNap1

**Fig. 4:**
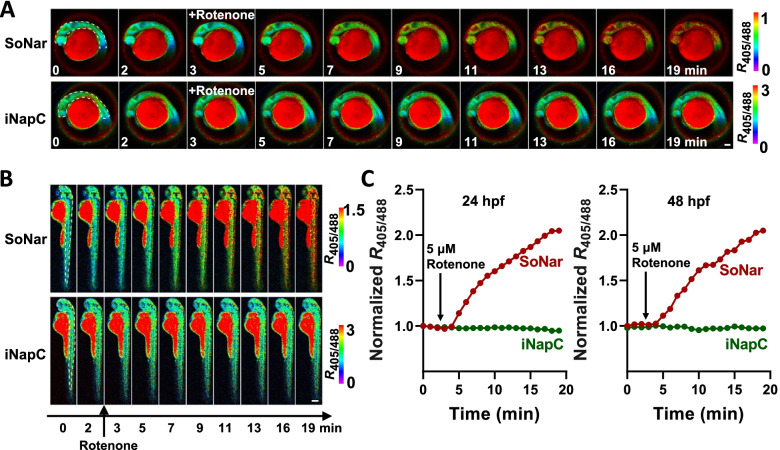
Monitoring NADH/NAD^+^ dynamics by SoNar in zebrafish embryos at 24 or 48 h post-fertilization. In vivo fluorescence imaging **A-B** and quantification **C** of zebrafish larvae (**A**, 24 hpf; **B**, 48 hpf) expressing SoNar or iNapC in response to 5 μM rotenone (mitochondria complex I inhibitor) indicating regions of interest (white dashed line). Highly viable wild-type AB zebrafish embryos were collected at one or two-cell stage and 1 nL sensor protein in HEPES buffer was injected into the animal pole. Embryos were collected and placed in a Petri dish with egg water (60 μg/mL Sea salts) at 28 °C. Larvae was anesthetized with 0.6 mM tricaine in E3 medium (5 mM NaCl, 0.17 mM KCl, 0.33 mM CaCl_2_, 0.33 mM MgSO_4_) and then imaged via a Leica TCS SP8 SMD confocal laser scanning microscope with a HCX Plan APO CS 10/0.40 NA or HC Plan FLUOTAR 5/0.15 NA dry objective. Zebrafish larvae in 48 hpf were dechorionated under the stereomicroscope using fine tweezers. All fluorescence images were pseudocolored by F_405_/F_488_ (R_405/488_). Scale bar, 100 μm **A** or 200 μm **B**. The handling procedures were approved by the Institutional Animal Care and Use Committee of Shanghai Institutes for Biological Sciences, Chinese Academy of Sciences. For **A-C**, adapted from the reference (Zou et al. 2018)

**Fig. 5:**
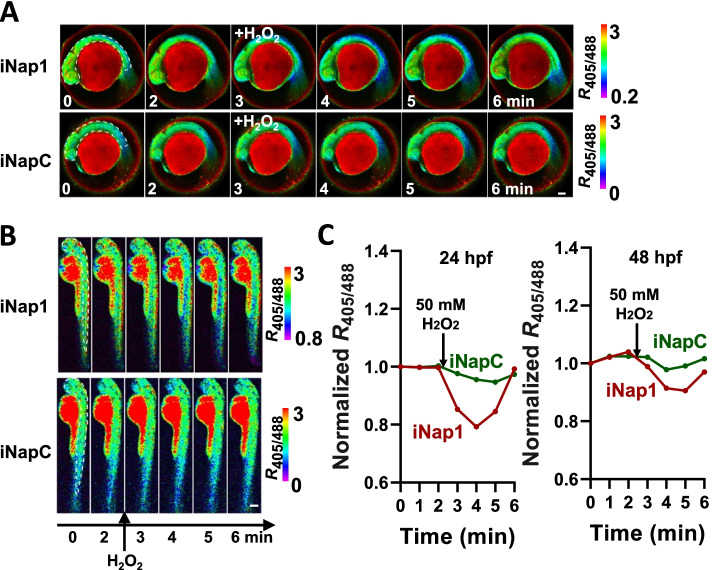
Monitoring NADPH dynamics by iNap1 in zebrafish embryos at 24 or 48 h post-fertilization. In vivo fluorescence imaging **A-B** and quantification **C** of zebrafish larvae (**A**, 24 hpf; **B**, 48 hpf) expressing iNap1 or iNapC in response to 50 mM H_2_O_2_ indicating regions of interest (white dashed line). The Procedures were similar to that in Fig. 4. Scale bar, 100 μm **A** or 200 μm **B**. For **A-C**, adapted from the reference (Zou et al. 2018)

*Danio rerio* is an excellent model organism for developmental research because its embryo is transparent and develops ex vivo. We chose zebrafish to establish and exemplify the application of sensors in studies of developmental metabolism. Both SoNar and iNap sensors have small molecular sizes and can be readily transferred into embryos through microinjection of sensor-coding DNA, mRNAs or purified sensor proteins. Using sensor nucleic acids allows for long-term and lineage-specific tracking, while using sensor proteins enables NAD(P)(H) monitoring immediately from fertilized eggs. For sensor proteins, less than ten nanograms of sterile protein is enough for microinjection into the animal pole of embryos at the one- or two-cell stage. For convenience of imaging, developing larvae are anesthetized with tricaine and dechorionated using tweezers. For imaging during the larval stage, melanization inhibitors such as N-phenylthiourea can be applied to prevent pigment formation. Fluorescence can be detected by a confocal laser scanning microscope system, and the use of supersensitive HyD hybrid detectors or light-sheet imaging systems should decrease phototoxicity. The majority of injected embryos develop into larvae without any noticeable abnormality. Fluorescence was relatively homogenous throughout the whole larval body, suggesting a uniform dispersion of sensor protein (Fig. 4A-B). Assays were carried out at either 24 h or 48 h after fertilization. The addition of 5 μM rotenone, a respiration complex I inhibitor, significantly increased the F405/F488 ratio of the SoNar sensor, whereas the treatment did not give rise to any response in the control fluorescent protein iNapC (Fig. 4C). Inhibition of mitochondrial respiration usually leads to a compensatory boosting of glycolysis and thus an elevation of cytosolic NADH/NAD^+^; therefore, this result verified that SoNar is able to monitor NADH/NAD^+^ dynamics in vivo in real time. Oxidative stress may perturb the NADPH pool by triggering cells to consume NADPH for survival. NADPH assays were also carried out at either 24 h or 48 h after fertilization (Fig. 5A-B). Hydrogen peroxide at 50 mM rapidly decreased the F405/F488 ratio of iNap1 within 1-2 min and then recovered quickly (Fig. 5C), suggesting rapid consumption and recovery of in vivo NADPH upon oxidative stress. The results show that mammalian cells have a strong tendency to maintain physiological NADPH homeostasis.

## Conclusions and perspectives

Differential gene expression is recognized as playing a central role in organismal development. Progression in sequencing technology, such as single-cell and spatial RNA sequencing, has led to an exciting flurry of epigenetic and transcriptomic studies on embryonic development (Mittnenzweig et al. 2021). Metabolic regulation of development, however, has been much less studied, which might be largely attributed to the great technological challenge of metabolite assays (Song and Shvartsman 2020). Namely, metabolites are not able to amplify in vitro, such as nucleic acids, and they usually demonstrate highly complicated spatiotemporal dynamics. Genetically encoded fluorescent sensors are a powerful tool for noninvasive metabolic monitoring. This technology achieves single-cell and subcellular resolution and real-time visualization of developmental processes; hence, it is much more cost-effective and time-saving than single-cell sequencing analysis. The biological insights that sensors give are also unique and thought-provoking. For example, lactate and other sensors have recently discovered a glycolytic burst that is induced by cytoskeleton remodeling during endothelial contraction, and sensor-based visible evidence is comparably convincing (Wu et al. 2021).

**Fig. 6:**
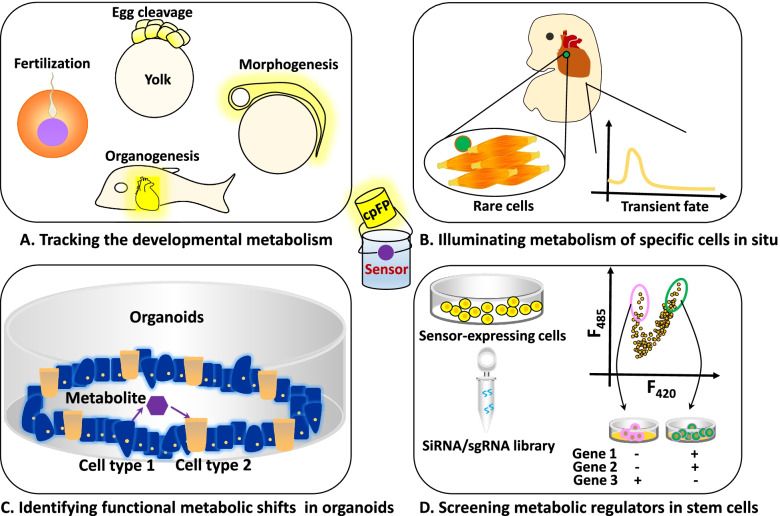
Typical applications of genetically encoded metabolite sensors in developmental research. **A** Real-time tracking live-cell or in vivo metabolism in different developmental stages such as fertilization, egg cleavage, morphogenesis and organogenesis. **B** Illuminating metabolism of specific cells (i.e. rare cells in developmental stages) in situ. **C** Identifying functional metabolic shifts in organoids. **D** Screening metabolic regulators in culture cells with siRNA or sgRNA library

Sensor-based assays have promising potential for developmental biology research. First, it can image cellular metabolism in situ during developmental and regeneration processes (Fall and Kumaran 2019) (Fig. 6A). The preparation and delivery of sensors can be readily realized, and imaging is particularly convenient for model organisms with ex vivo development, such as *Caenorhabditis elegans* and *Xenopus laevis* (Borodinsky 2017; Girard et al. 2007). To visualize mouse embryonic metabolism, a transgenic mouse line expressing a FRET pyruvate sensor was engineered, and embryos during the presomitic development stage were cultured ex vivo (Bulusu et al. 2017). The sensor reports an increase in glycolytic activity in the posterior presomitic mesoderm compared to the anterior mesoderm. Noticing genetic differences of model organisms and environmental factors such as temperature, codon usage and experimental protocols need to be optimized to ensure the correct soluble expression and fluorophore maturation of sensor proteins. Second, sensors can be employed to study the metabolism of rare cell types or transient developmental stages (Fig. 6B). It is of great challenge to acquire single-cell samples in these settings if single-cell analysis in vitro is applied (Chappell et al. 2018). However, cis-acting regulatory elements such as lineage- and stage-specific promoters and enhancers may be used to drive sensor expression in a controllable manner, enabling precise metabolic monitoring of cell types and stages of interest. Third, sensors may be applied to dissect how metabolism is coordinated with cellular signaling and gene expression control to form a functional organ using organoids (Fig. 6C). Recently, considerable progress has been made in organoid research to recapitulate the three-dimensional architectures and functions of organs (Kim et al. 2020). However, there are still many mysteries about the functions and underlying mechanisms of intracellular metabolites and intercellular communication. Take NAD^+^ as an instance. In adult mice, NAD^+^ can be synthesized from tryptophan in the liver and degraded into nicotinamide; then, nicotinamide is transported into other tissues for regeneration of NAD^+^(Liu et al. 2018). It is very intriguing to utilize organoids to investigate whether and how NAD^+^ levels are coordinated between different developing cells. Finally, stem cells are ideal systems in which sensors may have good applications. For example, using sensors as reporters, chemical and genetic screens can be readily carried out to identify and quantitatively analyze metabolic regulators in stem cells (Zhao et al. 2016b) (Fig. 6D).

Given the close link of NAD^+^/NADH and NADP^+^/NADPH, it is biologically important to decipher their circadian dynamics in subcellular compartments including cytosol, nucleus and mitochondria, and in different organs relevant to rhythmic regulations such as brain, liver and endocrine glands. Recently, we have developed a novel NAD^+^ metabolism sensor, named FiNad (Zou et al. 2020), and it allows us to integrate the redox toolbox (NAD^+^ sensor FiNad, NADH sensor SoNar, NADP^+^ sensor Apollo-NADP(+)(Cameron et al. 2016), and NADPH sensor iNap) to characterize the circadian oscillation landscape and functions of core coenzymes. Further research is necessary to address this issue.

To date, however, sensors for important metabolites are lacking, including most amino acids, intermediate metabolites in glycolysis, pentose phosphate pathway and TCA, nucleotides, lipids and vitamins, and the development of high-performance sensors is highly desirable. A limitation for sensor-based metabolic assays is that information on only one or at most a few metabolites can be acquired at one time; thus, combination with multiomics technologies is necessary for comprehensively understanding the genetic and metabolic control of organismal development.

## Data Availability

Not applicable.

## Abbreviations

FLIM: Fluorescence lifetime imaging
FRET: Fluorescent resonance energy transfer
NAD^+^: Oxidized nicotinamide adenine dinucleotide
NADH: Reduced nicotinamide adenine dinucleotide
NADP^+^: Oxidized nicotinamide adenine dinucleotide phosphate
NADPH: Reduced nicotinamide adenine dinucleotide phosphate
TCA: Tricarboxylic acid cycle
